# Analysis of the Determinants of Stunting among Children Aged below Five Years in Stunting Locus Villages in Indonesia

**DOI:** 10.3390/healthcare11060810

**Published:** 2023-03-09

**Authors:** Lasarus Atamou, Dwi Cahya Rahmadiyah, Hamidah Hassan, Agus Setiawan

**Affiliations:** 1 Department of Community Health Nursing, Faculty of Nursing, Universitas Indonesia, Depok 16424, Indonesia; lasarus.atamou@ui.ac.id (L.A.); dwicahyar@gmail.com (D.C.R.); 2 Department of Nursing, Faculty of Medicine and Health Sciences, Unversiti Tunku Abdul Rahman, Bandar Sungai Long, Kajang 4300, Selangor, Malaysia; hamidah@utar.edu.my

**Keywords:** stunting determinants, stunting locus village, children under five

## Abstract

Determinants of stunting are a concern in stunting locus villages, especially in East Nusa Tenggara, the province with the highest prevalence of stunting in Indonesia. This study aims to analyze the determinants of stunting in such villages. A cross-sectional research design was conducted on a sample of 166 mothers of children aged 24–59 months selected using a proportional random sampling method in four stunting locus villages in East Nusa Tenggara province, from January to March 2022. Chi-square and binary logistic regression were used to analyze the determinants of stunting with a significance level of *p* < 0.05. The prevalence of stunting among children aged below five years in the villages was 22.3%. Lack of maternal knowledge (AOR: 5.310; 95% CI: 0.671–41.997) and negative parenting (AOR: 3.026; 95% CI: 1.191–7.871) were associated with higher risk of stunting. Meanwhile, children aged below five years with close birth spacing (AOR: 0.304; 95% CI: 0.087–1.063) had a low risk of stunting. The prevalence of stunting in stunting locus villages needs special attention from the Indonesian government. Maternal knowledge should be enhanced by distributing information related to stunting through formal and non-formal education and teaching good parenting to reduce the prevalence of stunting among children aged below five years in stunting locus villages.

## 1. Introduction

Stunting is a global health problem and affects one or more among children aged below five years in 2020. This number continues to increase substantially due to constraints in accessing nutrition, diet, and other essential nutrition services during the COVID-19 pandemic [1]. Children under five are the most vulnerable to stunting. More than half of stunted children aged below five years globally (about 55%) come from Asian countries. According to World Health Organization (WHO), Indonesia has the third highest stunting prevalence in Southeast Asia [2,3,4]. Stunted children aged below five years have ages ranging from 0 to 59 months and have a nutritional status category with Z-score <−2 standard deviations according to the height-for-age index [5,6]. Stunting has multifactorial causes, and the immediate cause is inadequate long-term nutrition or frequent exposure to repeated infections. Long-term effects lead to reduced cognitive development, academic performance, increased disease risk, and poor future economic productivity. Stunting is expected to burden the national socioeconomic and health systems [7,8,9,10].

Based on the routine reports of the integrated SIGIZI (Nutrition Information System) and e-PPGBM (Electronic Recording and Reporting of Community-Based Nutrition), the target stunting rate for children aged below five years in Indonesia in 2020 is 24.1%; as of 20 January 2021, 11.6% of children aged below five years from 34 provinces are stunted, and the province with the highest stunting rate (24.2%) is NTT (East Nusa Tenggara). This information is in line with the results of the 2018 Riskesdas (basic health research) survey, which shows that NTT is the province with the highest stunting rate of 42.6%. Alor is one of the districts included in the top five with the highest prevalence of stunting (45.51%) in NTT province. Based on the Alor Regency government decree, this district has 21 stunting locus villages, and the Apui health center has the most locus villages [2,11,12].

Government policies to reduce the prevalence of stunting include Sustainable Development Goals (SDGs). The second goal of SDGs is to end hunger, achieve food security and improved nutrition, and launch sustainable agriculture. The second target of part A of SDGs is to end hunger, achieve food security and balanced nutrition, and address stunting in children. This situation follows Indonesia’s 2020–2024 RPJMN (National Medium Term Development Plan) policy, which raises one of the points related to stunting, namely, accelerating stunting reduction by increasing the effectiveness of specific and sensitive nutrition interventions. One of the stunting reduction intervention policies is the formation of stunting locus areas, including stunting locus villages. The stunting locus village is a village area in the stunting locus district that is determined by the government as the target focus of integrated stunting interventions by several sectors, namely, OPD (Regional Apparatus Organization), NGOs (non-governmental organizations), and the community, by holding a stunting convergence meeting to discuss how to reduce the incidence of stunting by integrating several sectors in a focused effort to reduce stunting. Despite several efforts of the Indonesian government to reduce the prevalence of stunting, the stunting prevalence rate in Indonesia remains at 14%, which is below the 2024 target [13,14,15]. In 2020, the Indonesian government targeted 260 stunting locus districts/cities to solve the stunting problem by organizing gathering activities. Stunting consultation aims to discuss the methods used by the government in consulting related to malnutrition. This practice is performed to ensure the integrated implementation of stunting reduction interventions between OPD in charge of services and sectors or non-governmental institutions from NGOs (non-governmental organizations) and community participants in all districts or cities up to the stunting locus villages [14,16,17,18].

Government and non-government efforts through specifics and sensitive nutrition interventions have not been able to achieve expected result to prevent stunting [19]. The purpose of this study was to describe the determinants of stunting and analyze the relationship between the determinants of stunting and the prevalence of stunting in children under five years of age in stunting locus villages that are the focus of stunting interventions by all policy makers in Alor Regency, East Nusa Tenggara Province.

## 2. Materials and Methods

### 2.1. Research Design

A cross-sectional research design was used to examine the determinants of stunting prevalence in children aged below five years in stunting locus villages. All variables observed were carried out simultaneously.

### 2.2. Data and Research Samples

The study was conducted from January to March 2022 in the population of mothers of children aged 24–59 months in the stunting locus village of Alor Regency, East Nusa Tenggara Province. A total of 238 mothers of children aged 24–59 months in the Apui health center working area were recruited using inclusion and exclusion criteria. Included in the inclusion criteria were: mothers who had children aged 24–59 months and had lived and settled in the stunting locus village area in the study area for at least one year; mothers who had children aged 24–59 months who were born at full term; and families who had more than one child, and were willing to participate in the study by signing informed consent; while the exclusion criteria were mothers who had children aged 24–59 months who had physical disabilities or had genetic disorders such as Down syndrome that could affect height measurements. A sample calculation was carried out using the Slovin formula. After obtaining the results, the results were divided into four stunting locus villages using proportional random sampling technique. The proportional random sampling technique was carried out by calculating the number of children aged 24–59 months in the stunting locus village, divided by the total number of children aged 24–59 months in the four stunting locus villages, then multiplied by the sample size obtained using the Slovin formula. The results of the sample calculation using this formula and the total sample using the proportional random sampling technique in the four stunting locus villages obtained a sample size of 166 children aged 24–59 months. Secondary data used to determine stunting in toddlers were obtained from the Apui Health Center based on the results of anthropometric measurements in February 2022.

### 2.3. Research Variables

The dependent variable is stunting status in children under five years of age. Stunting status variables are classified into stunting and non-stunting based on secondary data obtained from the results of anthropometric measurements based on height versus age by the Apui Health Center. The measurement results were adjusted to the standard deviation (SD) Z score value seen in the WHO growth curve standard deviation table. It is said to be stunted if the Z score value is <−2 SD and not stunted if the score is more or equal to −2 SD. The Z score values in the WHO growth curve standard deviation table are dichotomous data that show the results of calculations based on measurements of height versus age. The results of measuring height and age are continuous variables, which are then classified into stunting status into dichotomies, namely, stunting and non-stunting. The independent variables are gender, birth spacing, history of infectious diseases, maternal knowledge, maternal parenting, parental income, utilization of health services, and household sanitation. Gender variables were classified into male and female. The birth spacing variable was categorized into normal if the birth spacing was three years or more and closer if the birth spacing was less than three years. The infectious disease history variable was classified into often sick and rarely sick. Frequently sick if the children aged below five years had an infectious disease in the last six months with a minimum duration of three sick days, and rarely sick if the children aged below five years did not have an infectious disease in the last six months with a minimum duration of three sick days. Maternal knowledge variables were obtained by asking questions on a questionnaire related to the definition of stunting, factors that cause stunting, characteristics of stunting among children aged below five years, effect of stunting, and early detection of stunting. Maternal knowledge was classified into good, sufficient, and deficient: good if the total correct answers are 76–100%, sufficient if the total correct answers are 56–75%, and less if the total correct answers are ≤55%. Maternal parenting variables were classified into positive if the applied parenting was democratic and authoritarian, and negative if the parenting was permissive and neglectful. The parental income variable used Alor UMK (district minimum wage) standard, which is classified into the following: above the UMK and under the UMK. The health service utilization variable was measured based on the type of health services accessed during the last six months at health service facilities and classified into maximum, sufficient, and less. Household sanitation variables used healthy home assessment guidelines issued by the Ministry of Health with two classifications: a healthy environment if the score obtained is 1068–1200 and an unhealthy environment if the score obtained is <1068.

### 2.4. Data Analysis

The results were analyzed using SPSS version 21. Chi-square and binary logistic regression were performed to analyze data and interpret the results. AOR was used with 95% confidence interval (CI) and a significance level of *p* < 0.05.

### 2.5. Ethical Considerations

This study passed ethical review by the Faculty of Nursing Science at the Universitas Indonesia with Number: Ket-63/UN2.F12.D1.2.1/PPM.00.02/2022.

## 3. Results

Of the total 166 respondents, the majority of children aged below five years were male (52.4%). About 80.8% of the children aged below five years had normal birth spacing, and 68.7% were often sick. A total of 72.3% of the mothers had less knowledge about stunting, 67.5% had negative parenting, and 65.1% did not utilize health services. About 62.7% of the household sanitation for children aged below five years was unhealthy (Table 1).

**Figure 1:**
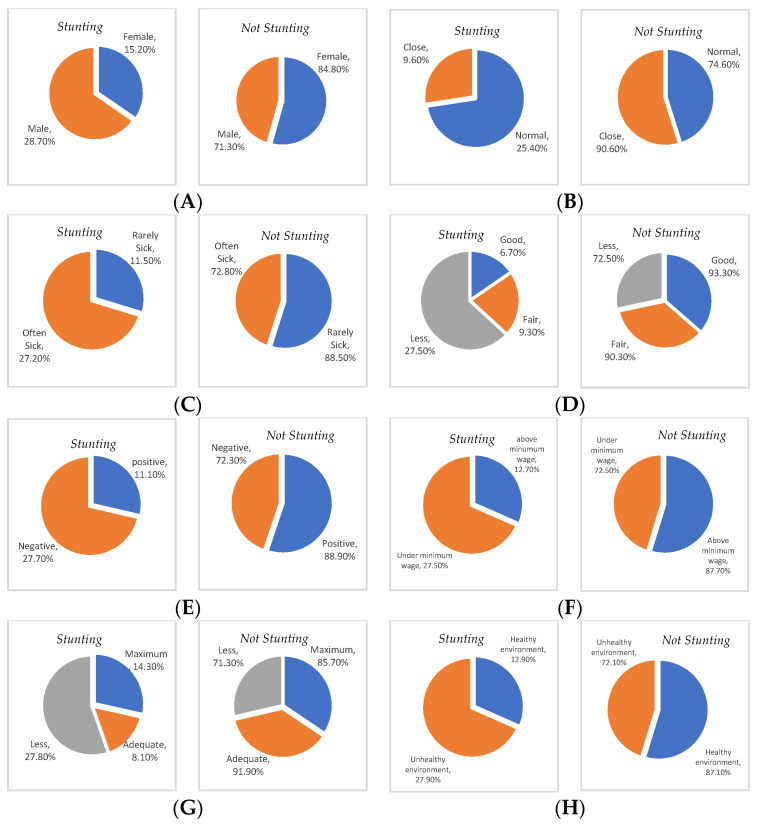
Pie chart of the distribution of stunting and non-stunting data in children aged below five years in the stunting locus village for (**A**) gender, (**B**) birth spacing, (**C**) history of infectious diseases, (**D**) maternal knowledge, (**E**) maternal parenting, (**F**) parental income, (**G**) utilization of health services, and (**H**) household sanitation.

Figure 1 explains the distribution of stunting prevalence based on the determinants studied, namely, gender, birth spacing, history of infectious diseases, maternal knowledge, maternal parenting, parental income, utilization of health services, and household sanitation.

Based on bivariate analysis, gender, birth spacing, history of infectious diseases, maternal knowledge, maternal parenting, parental income, utilization of health services, and household sanitation had a significant relationship to the prevalence of stunting among children aged below five years in the stunting locus village. Based on multivariate analysis, less maternal knowledge was associated with risk of stunting in children aged below five years by 5.31 times compared with good knowledge (AOR: 5.310; 95% CI: 0.671–41.997). The risk of stunting among mothers who applied negative parenting was 3.02 times that among mothers with positive parenting practices (AOR: 3.026; 95% CI: 1.191–7.871). The risk of stunting in children aged below five years who had a history of infectious disease, often sick, was 2.86 times that among children aged below five years, rarely sick (AOR: 2.863; 95% CI: 1.112–7.371). The risk of stunting in children aged below five years with parents’ income under minimum wage was 2.71 times that among children aged below five years with parents’ income above the minimum wage (AOR: 2.712; 95% CI: 1.108–6.643). The risk of stunting in children aged below five years who had household sanitation, unhealthy environment, was 2.61 times that among children aged below five years with healthy environment (AOR: 2.610; 95% CI: 1.107–6.151). The risk of stunting in children aged below five years who less utilized health services was 2.41 times that among children aged below five years with maximum utilization (AOR: 2.416; 95% CI: 0.664–8.788). The risk of stunting in male children aged below five years was 2.25 times that among female children aged below five years (AOR: 2.251; 95% CI: 1.042–4.863). The risk of stunting in children aged below five years with close birth spacing was 0.30 times that among children aged below five years with normal birth spacing (AOR: 0.304; 95% CI: 0.087–1.063). (Table 2)

## 4. Discussion

Reducing the prevalence of stunting involves cross-sectoral initiatives with various intervention programs both from the government and the community to reduce the prevalence of stunting. Data from basic health research in 2018 showed that the prevalence of stunting is still high, and achieving the 2024 national target of 14% requires implementation of appropriate interventions [17,20]. One of the interventions is the government’s effort to establish stunting locus villages as the focus of stunting interventions by all policyholders. Determinants of stunting in stunting locus villages are essential information in determining appropriate interventions to reduce the prevalence of stunting. Previous studies explained the determinants of stunting prevalence. However, no studies have been con ducted among stunting locus villages, especially in the province with the highest stunting prevalence rate in Indonesia, namely, NTT [14,21,22,23]. In the present study, several variables have a significant relationship with the prevalence of stunting in the stunting locus villages, consistent with previous works in stunting or non-stunting locus villages. In the stunting locus villages, the variables of maternal knowledge and parenting patterns were associated with higher risk for stunting in children aged below five years.

Maternal knowledge has a significant relationship to high risk of stunting. Lack of maternal knowledge can affect maternal attitudes and skills in caring for their children in the 1000 HPK period; examples are proper handling to prevent stunting in children aged below five years, improving nutritional status to achieve growth maturity, and determining maternal attitudes and behavior in providing food to meet nutritional adequacy and establish perspectives and other health actions for children aged below five years in the prevention of stunting [24,25]. Formal education obtained by mothers affects their knowledge about stunting. Maternal knowledge can help improve nutritional status in children to achieve growth maturity. Inadequate knowledge, a lack of understanding about good eating habits, and lack of understanding about stunting determine the attitude and behavior of mothers in providing food for their children, including the right type and amount so that children can grow and develop optimally. The higher the mother’s knowledge about stunting and health, the better the food assessment, while mothers with low knowledge often feed children without meeting their nutritional needs [26]. Good maternal knowledge provided a 6.7% contribution to the prevalence of stunting, and it contrasted with less maternal knowledge. Less maternal knowledge is higher at 27.5% and dominates the incidence of stunting in children aged below five years. Among the population aged 15–49 years in Alor District in 2020, 58.65% were married women, and 65.18% had a primary school education or below. In addition, among the population aged 15 years and older, 32.52% of the women had a primary school certificate, and 15.57% did not have a primary school certificate or did not attend or had dropped out of primary school [27,28,29]. The education level of the community, which affects the literacy rate, is still low, with 16.08% not having a primary school certificate and 31.53% having a primary school certificate. However, mothers with low levels of education can still have high knowledge about stunting, depending on their level of literacy in accessing knowledge through non-formal education.

This study also showed a significant relationship between maternal parenting and prevalence of stunting. Maternal parenting was associated with the second highest risk of stunting after maternal knowledge. Maternal parenting can affect the prevalence of stunting in toddlers because mothers have a very important role in regulating children’s food consumption patterns that are adjusted to the food sources available in the family. In addition, the mother is the closest person to the child since the child is a toddler, provides breast milk, provides food for the child’s growth and development, and takes care of everything when the child is sick. Good parenting inspires children to grow into adults with a good lifestyle. Therefore, good maternal parenting is very important to produce healthy children who are not stunted [30,31,32]. Positive parenting is the way parents treat their children, which can be recognized through the words and actions of parents that have a good impact on the development or independence of the child’s personality, especially in supporting stunting prevention. Positive parenting is shown in the types of democratic and authoritarian parenting, both of which support children in fulfilling nutritional needs and stunting prevention factors in children. Negative parenting is a parenting behavior that can be recognized through words and actions that have a negative impact on the development or independence of children’s personalities, especially in preventing stunting. Negative parenting is shown in the types of permissive parenting and neglect, where both parenting patterns do not support children in meeting nutritional needs and other factors preventing stunting in children. Maternal parenting is usually based on feeding, primary care, and hygiene and sanitation for children under five years old [31,33,34,35,36]. Negative maternal parenting had 11.1% higher contribution to the prevalence of stunting than positive parenting. Negative parenting is high because the community includes mothers of children aged below five years who are usually involved in gardening, so they tend to ignore the care of children aged below five years, and caring needs are left to the closest family. About 84.01% of the population in the study worked as garden farmers. In general, the parenting patterns of mothers to children aged below five years are permissive and neglectful. In the permissive type of parenting, parents are not strict in controlling children’s eating, so children tend to consume foods that do not contain adequate nutrition for their body needs. In the neglect type of parenting, parents tend to have farms in the garden, so childcare is neglected. Fulfillment of nutritional adequacy according to body needs is not considered, so children tend to experience chronic malnutrition, which can have an impact on stunting [33,34,35,37].

Birth spacing was significantly correlated with the prevalence of stunting but led to the lowest risk of stunting in children aged below five years in the stunting locus villages. A child’s birth spacing which is too close will affect the family’s nutritional status because the family has less optimal caring for the child. In addition, birth spacing of less than two years can lead to poor fetal growth, prolonged labor, and bleeding in labor because the uterus has not recovered properly, increasing the risk of anemia in mothers during pregnancy which has an impact on the risk of stunting in children [38,39,40]. In general, mothers in the stunting locus villages have normal birth spacing in children aged below five years. Each village has health workers who promote public awareness of using contraception. The presence of family planning extension workers in the sub-district who always routinely conduct socialization activities also contributes to normal birth spacing. In addition, NTT’s KIA (Maternal and Child Health) revolution program, which has the slogan that all pregnant women give birth in adequate health facilities, also contributes to normal birth spacing. In 2020, the percentage of women aged 15–49 years who have given birth with delivery assistance by health workers is 67.51%, so health workers provide good health postpartum counseling about birth spacing [27,41,42]

Male children aged below five years are more likely to experience stunting because it follows the typical pattern of dimorphism; boys tend to be heavier and taller than girls in areas with good health and nutritional status. However, in the stunting locus villages, the trend does not match the normal pattern of dimorphism. In the stunting locus village, it does not fit the normal pattern of dimorphism because stunted toddlers in the area experience a poor level of health and nutritional status [41,42,43]. In February 2022, a total of 53.62% of male children aged below five years were stunted in the stunting locus village of the public health center where the study was conducted. In addition to the pattern of dimorphism, one of the reasons why boys are more likely to experience stunting is because generally boys play away from home more often, so they tend not to get nutritious food at home because they are more focused on playing. In addition, 27.2% of the children aged below five years who are often sick tended to experience stunting more dominantly. Infectious diseases caused by pathogens are associated with inadequate water, sanitation, and hygiene (WASH) despite nutrition interventions. Despite nutritional interventions, but if WASH is inadequate, then it can risk causing stunting in children aged below five years. The most common contagious diseases are acute respiratory infections (ARI) and diarrhea [44,45,46]. In the stunting locus villages, children aged below five years commonly experience ARI due to the habit of playing together, leading to easy transmission of ARI. In addition, ARI is also one of the infectious diseases that can be easily infected in children, especially in children with stunting. Children aged below five years with parental income under the minimum wage are more likely to experience stunting because it is related to family purchasing power. One of the indirect causes of stunting is influenced by socioeconomic aspects. Economic factors are one of the factors that indirectly affect stunting rates in children aged below five years. Low economic status has an impact on the lack of food availability and quality due to low family purchasing power [3,47,48]. Efforts to improve the economy and family income, for example, by preparing market facilities to support the economy, need to be made because, in this study, it is known that the market is located around the locus village with difficult road conditions, making it difficult to distribute economic goods to be sold to increase family income. Family income above the minimum wage can increase the purchasing power of food to prevent stunting [27,41,47]. In addition, mothers who did not utilize health services had higher prevalence of stunting among their children aged below five years. With regard to children aged below five years with maximum utilization of health services, their growth and development processes are monitored, and their health status is considered, in order to reduce the risk of stunting. Based on data obtained from the primary health care, it is known that in the stunting locus village, there is a health service post that has been placed by health workers, but the tendency to take children aged below five years to access health service facilities is still very minimal. Reaching the nearest health facility in the stunting locus village is difficult [26]. Integrated service post activities that are usually carried out regularly, i.e., once a month, are also sometimes not attended by mothers and children aged below five years due to other activities, for example, mothers who have been in the garden since the morning. In addition, there are some mothers who, although they know the schedule of integrated service post activities, lack the interest to utilize these activities. Mothers of children aged below five years assume that their children are healthy, so it is not too important to be taken to integrated service post activities; children aged below five years are taken to a health facility only if they are sick. The view and assumption that health facilities are only accessed when toddlers are sick is still a habit of mothers of children aged below five years, while this is clearly not in accordance with the healthy living community movement program launched by the Ministry of Health, one aspect of which is to check health regularly both in healthy and sick conditions. Generally, the community assumption that health facilities are only accessed when children aged below five years are sick is still a habit that contributes to the high prevalence of stunting in the stunting locus villages [3,49,50]. Unhealthy household sanitation factors tend to dominate stunting in children aged below five years. The prevalence of stunting will decrease if household environmental health is good [22,46,51,52,53,54]. The results of the study are in line with the subjective sanitation data of the Puskesmas where the study was conducted, showing that most household environments were unhealthy because the sanitation facilities were generally inadequate and did not meet the health requirements, with conditions such as dirt floors, lack of windows and ventilation, and lack of ceilings. Housing health requirements include three aspects of assessment: the house component group, the sanitation facility group, and the occupant behavior group. In general, the three assessment groups are inadequate to support stunting prevention [55].

### 4.1. Limitations of the Study

This study was limited to the stunting locus village in the working area of the Apui health center, Alor district, NTT province. The results cannot be generalized to other stunting locus areas because each area has its own health status of toddlers, health services, parenting and feeding practices, and sanitation levels that can affect the incidence of stunting. In addition, socioeconomic differences and education level in each region are the limitations of this study. Therefore, data obtained are only specific to the stunting locus village of the Apui community health center. Nevertheless, government programs for stunting intervention in Indonesia have similarities.

### 4.2. Suggestion

Determinants of stunting prevalence among children aged below five years in the locus village provided information for policyholders in planning interventions to reduce stunting prevalence. Interventions to improve maternal knowledge and good parenting, as well as health education about stunting to mothers of children aged below five years, are essential to reduce the prevalence of stunting in children aged below five years in stunting locus villages. In addition, household sanitation, economic and health factors of children aged below five years are important to consider to develop appropriate interventions to prevent stunting among children aged below five years in the stunting locus villages.

## 5. Conclusions

This study analyzed the relationship of determinants of stunting prevalence in children aged below five years in stunting locus villages. Gender, birth spacing, history of infectious diseases, maternal knowledge, maternal parenting, parental income, utilization of health services, and household sanitation have a significant relationship with the prevalence of stunting. Maternal knowledge and maternal parenting are associated with the highest risk of stunting, and birth spacing is related to the lowest risk. Increasing maternal knowledge and parenting through health promotion and distribution of health information must be implemented to reduce the prevalence of stunting in children aged below five years in stunting locus villages.

## Figures and Tables

**Table 1:** Determinants of stunting (n = 166).

Variable	n	%
Gender		
Female	79	47.6
Male	87	52.4
Birth spacing		
Normal	134	80.7
Close	32	19.3
History of infectious disease		
Rarely sick	52	31.3
Often sick	114	68.7
Maternal knowledge		
Good	15	9.0
Fair	31	18.7
Less	120	72.3
Mother’s parenting pattern		
Positive	52	32.5
Negative	112	67.5
Parents’ income		
Above minimum wage	57	34.3
Under minimum wage	109	65.7
Health service utilization		
Maximum	21	12.7
Adequate	37	22.3
Less	108	65.1
Household sanitation		
Healthy environment	62	37.3
Unhealthy environment	104	62.7

**Table 2:** Bivariate and multivariate analyses of the determinants of stunting among children aged below five years in stunting locus villages.

Variable	Stunting				X^2^	AOR	95% CI	
	No		Yes					
	n	%	n	%			Lower	Upper
Gender								
Female	67	84.8	12	15.2	4.368 **	Ref		
Male	62	71.3	25	28.7		2.251	1.042	4.863
Birth spacing								
Normal	100	74.6	34	25.4	3.817 **	Ref		
Close	29	90.6	3	9.6		0.304 **	0.087	1.063
History of infectious disease								
Rarely sick	46	88.5	6	11.5	5.052 **	Ref		
Often sick	83	72.8	31	27.2		2.863	1.112	7.371
Maternal knowledge								
Good	14	93.3	1	6.7	26.841 ***	Ref		
Fair	28	90.3	3	9.3		1.500	0.143	15.765
Less	87	72.5	33	27.5		5.310 ***	0.671	41.997
Mother’s parenting pattern								
Positive	48	88.9	6	11.1	25.774 ***	Ref		
Negative	81	72.3	31	27.7		3.026 ***	1.191	7.871
Parents’ income								
Above minimum wage	50	87.7	7	12.7	5.020 **	Ref		
Under minimum wage	79	72.5	30	27.5		2.712	1.108	6.643
Health service utilization								
Maximum	18	85.7	3	14.3	7.638 **	Ref		
Adequate	34	91.9	3	8.1		0.529	0.097	2.896
Less	77	71.3	31	27.8		2.416	0.664	8.788
Household sanitation								
Healthy environment	54	87.1	8	12.9	5.033 **	Ref		
Unhealthy environment	75	72.1	29	27.9		2.610 *	1.107	6.151

*** *p* < 0.01, ** *p* < 0.005, * *p* < 0.1 AOR: Adjusted Odd Ratio, CI: Confidence Interval, X^2^: Chi-Square.

## Data Availability

The data presented in this study are available on request from the corresponding author.
